# Probabilistic Identification of Cerebellar Cortical Neurones across Species

**DOI:** 10.1371/journal.pone.0057669

**Published:** 2013-03-04

**Authors:** Gert Van Dijck, Marc M. Van Hulle, Shane A. Heiney, Pablo M. Blazquez, Hui Meng, Dora E. Angelaki, Alexander Arenz, Troy W. Margrie, Abteen Mostofi, Steve Edgley, Fredrik Bengtsson, Carl-Fredrik Ekerot, Henrik Jörntell, Jeffrey W. Dalley, Tahl Holtzman

**Affiliations:** 1 Computational Neuroscience Research Group, Laboratory for Neuro- en Psychophysiology, K.U. Leuven School of Medicine, Leuven, Belgium; 2 Department of Otolaryngology, Washington University, St. Louis, Missouri, United States of America; 3 Department of Neurobiology, Washington University School of Medicine, St. Louis, Missouri, United States of America; 4 The Division of Neurophysiology, The National Institute for Medical Research, London, United Kingdom; 5 Department of Neuroscience, Physiology and Pharmacology, University College London, London, United Kingdom; 6 Department of Physiology, Development and Neuroscience, University of Cambridge, Cambridge, United Kingdom; 7 Department of Experimental Medical Science, Section for Neuroscience, Lund University, Lund, Sweden; 8 NeuroNano Research Center, Lund, Sweden; 9 Department of Psychiatry, University of Cambridge, Addenbrooke's Hospital, Cambridge, United Kingdom; 10 Department of Psychology, Behavioural and Clinical Neuroscience Institute, University of Cambridge, Cambridge, United Kingdom; Tokyo Medical and Dental University, Japan

## Abstract

Despite our fine-grain anatomical knowledge of the cerebellar cortex, electrophysiological studies of circuit information processing over the last fifty years have been hampered by the difficulty of reliably assigning signals to identified cell types. We approached this problem by assessing the spontaneous activity signatures of identified cerebellar cortical neurones. A range of statistics describing firing frequency and irregularity were then used, individually and in combination, to build Gaussian Process Classifiers (GPC) leading to a probabilistic classification of each neurone type and the computation of equi-probable decision boundaries between cell classes. Firing frequency statistics were useful for separating Purkinje cells from granular layer units, whilst firing irregularity measures proved most useful for distinguishing cells within granular layer cell classes. Considered as single statistics, we achieved classification accuracies of 72.5% and 92.7% for granular layer and molecular layer units respectively. Combining statistics to form twin-variate GPC models substantially improved classification accuracies with the combination of mean spike frequency and log-interval entropy offering classification accuracies of 92.7% and 99.2% for our molecular and granular layer models, respectively. A cross-species comparison was performed, using data drawn from anaesthetised mice and decerebrate cats, where our models offered 80% and 100% classification accuracy. We then used our models to assess non-identified data from awake monkeys and rabbits in order to highlight subsets of neurones with the greatest degree of similarity to identified cell classes. In this way, our GPC-based approach for tentatively identifying neurones from their spontaneous activity signatures, in the absence of an established ground-truth, nonetheless affords the experimenter a statistically robust means of grouping cells with properties matching known cell classes. Our approach therefore may have broad application to a variety of future cerebellar cortical investigations, particularly in awake animals where opportunities for definitive cell identification are limited.

## Introduction

Obtaining reliable assignments of spike discharges to identified neuronal types *in vivo* is a major problem, particularly in awake behaving animals [1]. Amongst the sensorimotor areas of the brain, the cerebellum offers a tractable circuit to study owing to its few well-defined cell-types. However, only Purkinje cells can be definitively identified using their unique responses to climbing fibre inputs [2]. Previous studies have employed a variety of measures based on spike timing or waveform characteristics to tentatively classify other neurone types [3]–[5], in some cases supported by juxtacellular labelling [6]–[9], or intracellular staining and/or assessment of membrane properties [10]–[12]. Anaesthetised animals have been widely used as they can provide a ground-truth through neuronal labelling although this is much harder to achieve in awake animals where spike-shape or firing-pattern derived measures tend to be relied upon. Spike-waveform shapes have been used in the cerebellum [4], [5], [13] and also in frontal cortex [14], barrel cortex [15] and ventral striatum [16]. Whilst spike-shapes carry potentially useful information for classifying neuronal classes, they can vary with electrode type and the geometric relationship between the electrode and the spike generation zone [17], [18]. Moreover, spike-shape measurement is achieved with a variety of techniques, making it difficult to compare and standardise between laboratories.

The heterogeneous morphological, neurochemical and synaptic connectivity of cerebellar interneurones [19], [20] is expected to impart distinctive firing patterns to the different classes of local interneurones. The recent use of a C4.5 decision-tree algorithm (a popular version of an algorithm to build a decision tree [21]) to classify local interneurones, within a restricted part of the cerebellum (vestibulocerebellum), using spontaneous activity signatures [9] lends weight to this viewpoint. However, decision-tree algorithms result in orthogonal decision boundaries, leading to inferior results with correlated parameters such as firing rate and irregularity. The method also requires numerous decision-steps, applied in a specific order and does not provide a measure of confidence surrounding the final decision. Here, we use a probabilistic approach (Gaussian Process Classifier) to classify cerebellar granular layer neurones, molecular layer neurones and Purkinje cells using firing rate and irregularity metrics.

Driven by the anatomical distinction between the granular and the molecular layers of the cerebellar cortex, we assessed the usefulness of a GPC-based approach for classifying neurones in each of these layers. Custom-built GPC models for the granular and molecular layers achieved 99.2% and 92.7% accuracy, respectively. In a cross-species comparison, using identified neurones the same approach achieved 80–100% accuracy using data drawn from anaesthetised mice and decerebrate cats. Based on the high levels of accuracy in mice, rats and cats, we assessed unidentified data from awake rabbits and monkeys and used our GPC to identify subsets of cells bearing the closest similarity to identified cell classes. Our approach highlights an extensive consistency of neuronal firing patterns between species and between behavioural 'states', implying a broad applicability of our GPC model to awake animal experiments.

## Materials and Methods

All procedures were conducted in accordance with the relevant national laws relating to animal use for scientific research and approved by the University of Cambridge Ethical Review Panel (rats and rabbits), by the University College London Animal Ethics Committee (mice), by the Malmö/Lund Animal Research Ethics Committee (permit number and approval-ID: M32-09) at the University of Lund (cats) and Washington University (primates). Methods as well as general animal care and welfare regarding the treatment of primates in our research conformed to the National Institute of Health (NIH) Guide for the Care and Use of Laboratory Animals and were approved by the Washington University Institutional Animal Care and Use Committee. Animals are housed in individual cages or in pairs with sufficient space for exercising following NIH guidelines. Animals are given primate food (pellets), fruits and food-treats twice a day. Additional fruits and food-treats are provided after each experimental session. Analgesics were used when directed by veterinarians to prevent pain of discomfort after surgery. A strong environmental enrichment program is active in Washington University to provide toys and pair animals for social interaction.

**Figure 1:**
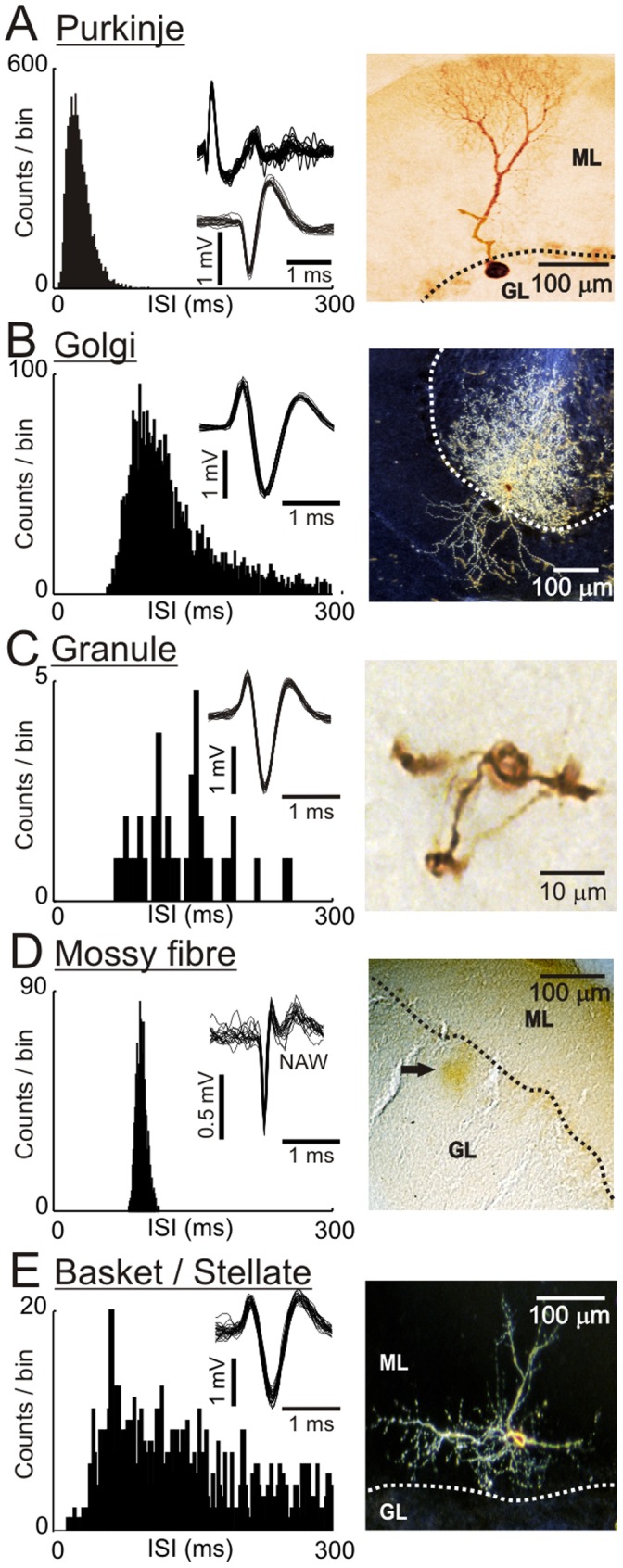
Activity patterns of cerebellar cortical cells in the rat. **A** shows an ISIH and spike-shapes (band pass 0.3–10 kHz) from an example Purkinje cell - note the presence of two spike-shapes, complex (top) and simple (bottom). The right panel shows a bright-field micrograph of a Purkinje cell following juxtacellular labelling with neurobiotin - note the characteristic dendritic arbor in the molecular layer (ML). **B–E** follow the same format for an example Golgi cell, granule cell, a regular firing mossy fibre terminal and basket/stellate cell, respectively. Although each of these granular layer units have broadly similar mean firing rates (compare the ISIHs), their intrinsic irregularities are divergent. Note that the spike-shapes shown for the Golgi cell, granule cell and basket/stellate cell are highly similar due to being recorded in the juxtasomatic configuration, whilst the spike-shape for the mossy fibre terminal is composed of an early fast and later variable negative after wave (NAW). The micrographs show a typical Golgi cell, viewed in dark-field, with dendrites extending into the ML and profuse, highly arborised axon tree in the granular layer (GL: note GL dendrites are not visible). In contrast, the much smaller granule cell shown in bright-field has a soma with three short dendrites. The micrograph in **D** shows a neurobiotin deposit in the upper granular layer following a juxtacellular labelling attempt with a regular firing mossy fibre unit (indicated by the arrow). Example data from a basket/stellate cell are shown in **E**, with a cell visible in the lower third of the molecular layer (ML) with arborisations extending in the parasagittal plane and presumed dendrites ascending in the plane of the Purkinje cell dendrites. Micrographs shown in **A** & **B** reproduced with permission from [7].

Our datasets consisted of neuronal recordings made in anaesthetised and decerebrate preparations: rat (urethane [7], [22]); mouse (ketamine/xylazine [24]); cat (decerebrate [10], [23]). In these preparations, efforts were made to elucidate cellular identities using intracellular recording and assessment of membrane biophysics (mouse) or intracellular/juxtacellular labelling (rat & cat), see examples in Figure 1. We also used data obtained from awake animals; macaques (unpublished data) and awake rabbits [25] (see original studies for details on animal preparation). Recordings of neuronal activity were obtained from Purkinje cells, molecular layer units and granular layer units in the absence of overt stimulation, thus such activity is considered spontaneous; for awake animals, periods of quiet rest were used. In the awake animal datasets, only Purkinje cells could be definitively identified due to the presence of complex spike discharges [2], whilst the remainder of the units sampled were considered to have been recorded in the granular layer (see original studies for details), therefore their identification remains as putative.

Using the rat dataset, we employed a Gaussian Process Classifier (GPC) [26] to infer the probability of a given cell belonging to a particular cell class. Spike trains in our rat dataset (used to build our GPC model) had a variety of lengths ranging from a minimum of 65 spikes to 13650 spikes, dependent on the firing rate of the neurone under study. For Golgi cells we had on average ∼1400 spikes, whereas for granule cells, regular firing mossy fibre terminals, Purkinje cells, stellate and basket cells we had on average ∼680, ∼1500, ∼5500, ∼1038 and ∼1014 spikes, respectively. The GPC is realized by maximizing a strict lower bound on the marginal likelihood of a multinomial probit regression model. The GPC developed in [26] is a variational Bayesian approach for multi-class Gaussian process classification. We used the radial basis function (RBF) as the Gaussian process (GP) covariance function. The (i,j) th element of the covariance matrix **C** in case of the RBF is defined as 
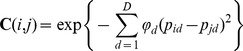
, where p_id_ is the d-th parameter extracted from the spike train (e.g. entropy) for cell ‘i’, D is the number of parameters used and 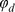 a covariance function hyperparameter for dimension d. One of the appealing properties of the GPC developed in [26] is that the 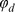 can be inferred from the data using importance sampling. Importance sampling is a technique for estimating parameters of a particular distribution, while only having samples generated from a different distribution rather than the distribution of interest, because parameter estimation is too hard to treat analytically, see [26].

The covariance matrix C is of dimension N×N where N is the number of cells: N equals 120 cells for building the GPC model of the granular layer and 41 cells for building the GPC model of the molecular layer.

**Figure 2:**
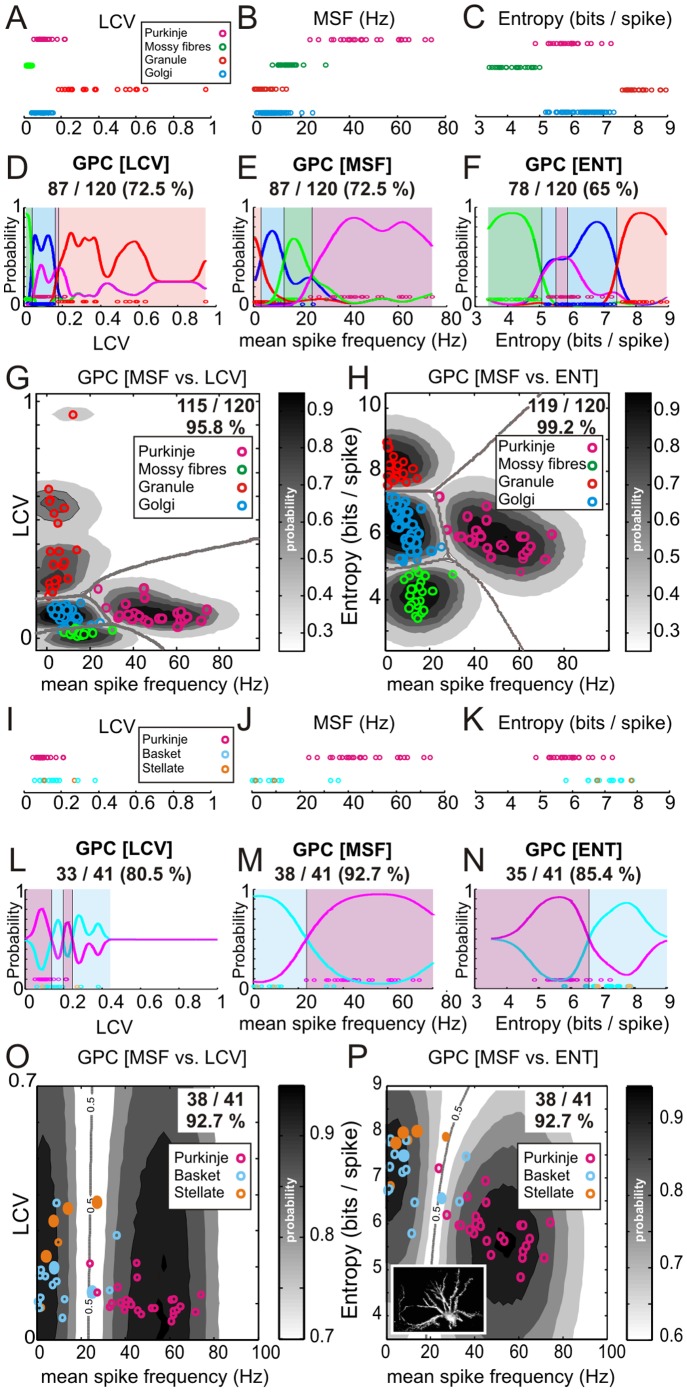
Firing frequency and firing irregularity measures for granular layer and molecular layer neurones. Three of the most useful statistics, CV of log ISIs (LCV), mean spike frequency (MSF) and log-interval-entropy (ENT), for classifying cells into distinct classes are plotted in **A–C** for the granular layer cells and **I–K** for the molecular layer cells. Each circle represents the spike train statistic of a single neurone (n = 120 cells and 41 cells, respectively). These data were used to build Gaussian Process Classifiers (**D–F** and **L–N**, respectively) using a single statistic to infer probabilities for each neurone belonging to a class and to delineate equiprobable decision boundaries between cell classes (solid black lines). Classification accuracy for each statistic is provided above each plot. **G** & **H** show the outcome of Gaussian Process Classifiers built using a twin-variate approach for the granular layer cells and **O** & **P** show the same analysis for the molecular layer cells (note inset labelled basket/stellate cell [cat]). Probability contours are superimposed for each class (probability levels = 0.25, 0.4, 0.6, 0.7, 0.8, 0.9 & 0.95) as well as the 2-dimensional decision boundaries and a resultant increase in classification accuracy (included in top right of each panel).

**Figure 3:**
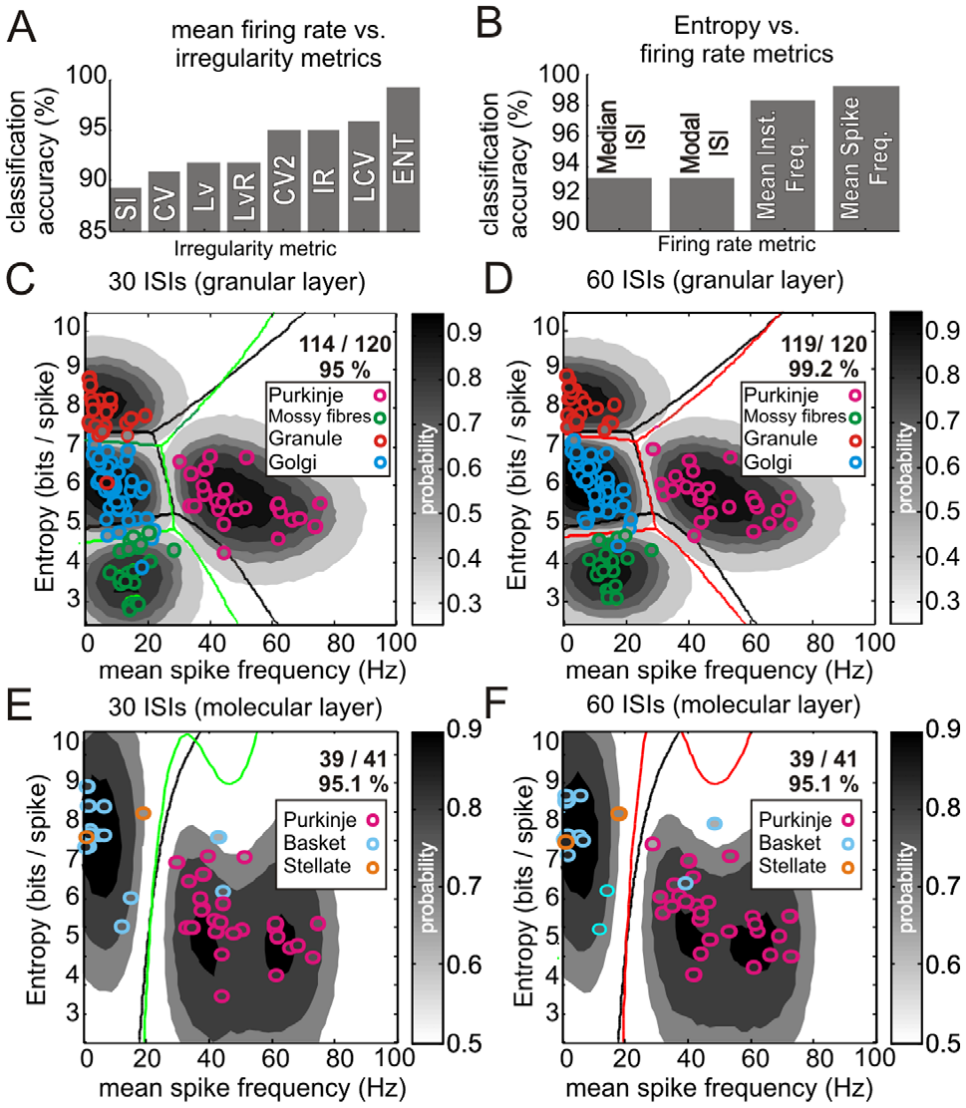
Comparison of irregularity measures, frequency measures and spike-train length on classification accuracy. The bar chart shown in **A** plots the Gaussian Process Classifier LOO-CV accuracy for our rat dataset using a range of firing irregularity statistics combined with MSF. The worst combination was MSF vs SI offering 89% classification accuracy, whilst MSF vs. ENT performed at 99.2% accuracy. **B** shows a similar analysis with a variety of frequency measures combined with ENT. Median and Modal ISI offered ∼93% accuracy with a marginal difference between mean instantaneous frequency and mean firing rate (98% and 99.2%, respectively). **C** & **D** show Gaussian Process Classifiers built on the granular layer dataset but with 30 ISIs or 60 ISIs, respectively. The decision boundaries for the model built on all spikes are superimposed (black lines), along with the recomputed decision boundaries (green and red lines respectively). Note that the probability contours are specific to each model. Using 30 ISIs offered a prediction accuracy of 95% whilst the model built with 60 ISIs for all cells offered the same accuracy as the all-spikes model (99.2%; c.f. Figure 2H). This shows the model can be built and applied to spike trains containing as little as 60 ISI’s without a decrease in performance. This allows a prediction in the order of a few seconds for the slowest firing neurones (granule cells). **E** & **F** show Gaussian Process classifiers built on the molecular layer dataset following the same convention as above. Note that the probability contours are specific to each model. Using 30 or 60 ISIs offered prediction accuracy of 95.1% in both cases, comparable to the all-spikes model (92.7%; c.f. Figure 2P).

**Figure 4:**
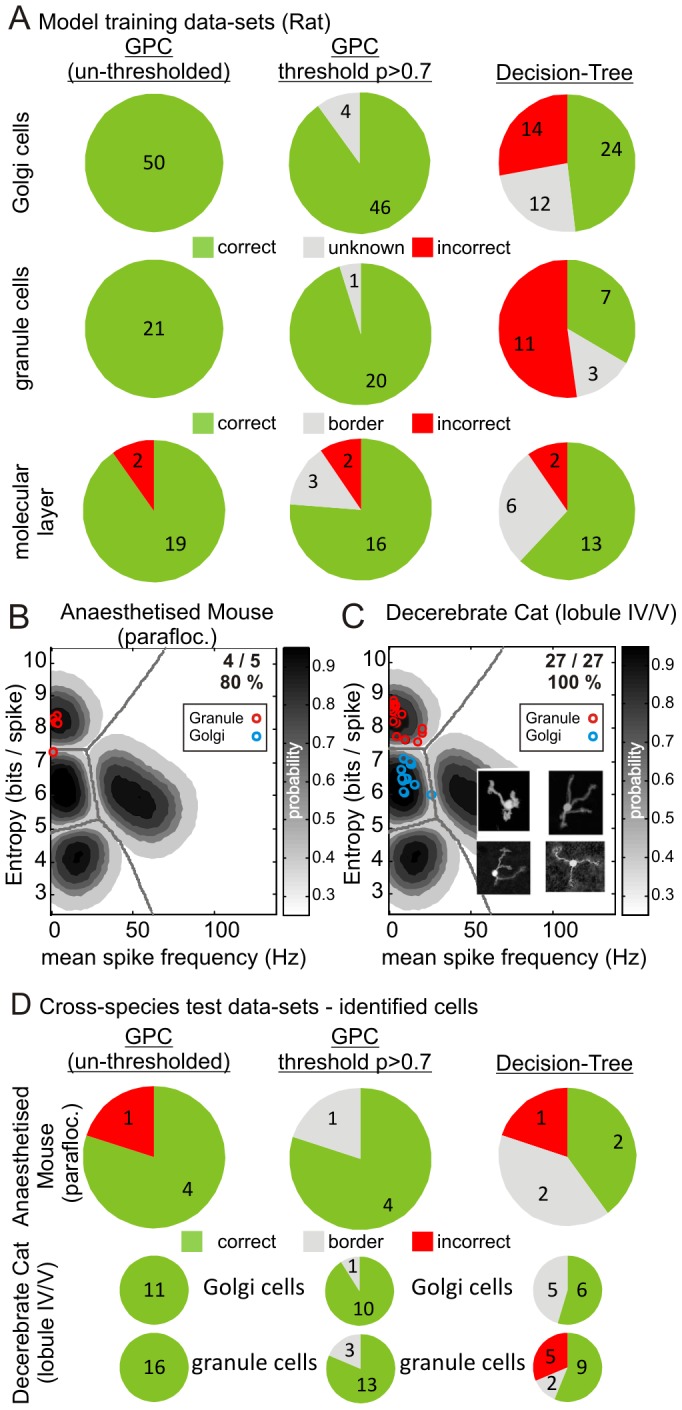
Decision-tree algorithm performance with identified neurones across mice, rats and cats. Following the format used by Ruigrok *et al.* (2011), **A** compares the classifications our of GPC model with those of the decision-tree algorithm. The *left* and *middle* columns show the outcomes of our GPC classification taking all cells without probability-thresholding (*left*) or with an arbitrarily chosen probability threshold of (p>0.7; *middle* column) thus some cells were classified as 'unknown' cells (c.f. the Ruigrok decision-tree outputs). The *right* column shows the results of our datasets when classified using the Ruigrok decision-tree. The numbers within the pies indicate the number of cells that were classified correctly, incorrectly and as 'unknown' cells (i.e. cells for which no decision is taken). In general, the decision-tree algorithm was less accurate than our GPC model, although note that neither mossy fibres nor Purkinje cells were built in to the decision-tree algorithm. **B** shows data from a small set of identified granule cells recorded in the anaesthetised mouse, leading to 80% classification accuracy. **C** shows similar data for identified granule cells (inset shows confocal reconstructions of juxtacellularly labelled granule cells) and Golgi cells recorded in the decerebrate cat, leading to 100% classification accuracy. Following the same format as in **A**, we compare mouse and cat data using our GPC model and the decision-tree algorithm. Generally lower levels of accuracy were achieved by the decision-tree algorithm.

We used the leave-one-out cross-validation (LOO-CV) technique [27] to estimate the accuracy of prediction on the rat data. This validation is closest to our objective of using the 120 rat cells from the granular layer model to make predictions for new cells as we demonstrated for the cells of the mouse, cat, rabbit and monkey. Similarly this validation is closest to using the 41 neurones of the molecular layer model to make predictions for new cells as demonstrated. In the leave-one-out procedure a GPC is built using all cells except for one cell that is left out (hence N = 119 or N = 40). The probability of that cell to belong to each of the cell classes is then computed based on the classifier. The cell is then assigned to the class with the highest probability according to Bayes’ decision rule. The prediction of the model can then be compared with the known cell type to verify whether the model made a correct decision. This procedure is repeated over all cells, so that each cell has been tested once. The final classification accuracy is then reported as the percentage of cells that were classified correctly. In case of the granular layer model, a GPC was built on all rat cells (hence N = 120) and the probability for each cell of the other species to belong to the different cell classes was inferred. For the molecular layer model a GPC was built on a mixture of anaesthetized rat cells (stellate cells and Purkinje cells) and decerebrate cat cells (stellate and basket cells). The cells are then assigned to the class which has the highest probability. The percentages of cells that were correctly classified are reported as the classification accuracies. The GPC models and decision boundaries in Figure 2 D–H, 3 C–D, 4 B–C, 5 A, C & E were built when using all 120 available cells of the granular and Purkinje layers. The GPC models in Figure 2 L–P, 3 E–F were built when using 15 stellate/basket cells and the 26 available Purkinje cells. Note that in Figure 2H all cells fall at the correct side of the decision boundary and therefore it appears that no error is made, however the reported accuracy of 99,2% (119/120) is based on our LOO-CV in which not all cells were used to build models (described earlier).

## Results

In this study we use a large dataset consisting of spontaneous activity of Purkinje cells and a variety of granular layer units, recorded in Crus I/II in anaesthetised rats, as well as molecular layer units, recorded in Crus I/II in anaesthetized rat and lobule IV/V of the decerebrate cat. As shown in Figure 1, Purkinje cells are the most easily identified due to their unique dual discharge of complex spike-waveforms [2] alongside simple spikes (Figure 1A, left panel). An example Purkinje cell is shown following juxtacellular labelling with neurobiotin (Figure 1A, right panel) revealing a typical soma located in the Purkinje cell layer (dotted line) and dendrites extending into the molecular layer (ML).

In contrast to Purkinje cells, several authors have described characteristic discharges assumed to be Golgi cells; large neurones located in the granular layer of the cortex. In the rat, at rest these cells are active with relatively low firing rates 2–25 Hz [7], [22], [28], 1.9–11 Hz [9], broad peaked inter-spike interval histograms (c.f. Figure 1B, left panel), a scarcity of intervals less than 30 ms and tuning distances of 50–150 µm with generally broad action potential shapes [4]–[9], [29]–[32]. An example Golgi cell following juxtacellular labelling is shown in Figure 1B (right panel) - note the dendrites extending in to the ML and the highly arborised axon within the granular layer (GL; note that GL dendrites are not visible in this micrograph).

Fewer studies have addressed the activity of granule cells *in vivo*; at rest, typically they are inactive or characterised by generally irregular firing patterns sometimes punctuated by 'bursts' of activity but often with long periods without discharge [6], [9]–[11], [22]–[24]. An inter spike interval histogram (ISIH) and photomicrograph of an example juxtacellularly labelled granule cell is shown in Figure 1C. Note the similarity of the spike-shape to the Golgi cell (c.f. Figure 1B) - in each case spikes were recorded in the juxtacellular configuration, with an initial positive deflection in the spike-shape - this component 'grows' as the electrode advances into the juxtacellular configuration [7], [17]. In anaesthetised rats, granule cells commonly respond with long-lasting excitations which are causally related to the long-lasting depression responses seen in their counterpart inhibitory Golgi cells [22].

Mossy fibres are present throughout the granular layer, with each of multiple mossy fibre rosettes contacting 50–150 granule cells whilst each granule cell receives roughly four mossy fibre inputs [33], [34]. Mossy fibre firing patterns can appear similar to those of granule cells, characterised by irregular activity punctuated by 'bursts' [6], [12], [23], [32] however, comparison of mossy fibre firing patterns is not straightforward since they relate to the source and modality of information they convey [12], [23]. In the rat Crus I/II, where our recordings were made, we commonly isolated units with highly rhythmic firing patterns. The spike-shapes of these units comprise an early fast- and later variable slow-component typical of mossy fibre terminals [4], [10], [32], [35]. At rest, these units are spontaneously active with mean firing rates ranging from ∼7–22 Hz, overlapping with Golgi cell firing rates (Figure 1D). Although we could not juxtacellularly label these units, an example labelling attempt resulting in a neurobiotin deposit in the granular layer is shown in the photomicrograph in Figure 1D.

The molecular layer contains two types of inhibitory interneurones; basket cells and stellate cells. These two cell types are distinguished principally by their depths within the molecular layer, basket cells residing in the deepest one third and stellate cells within the outer two thirds of the molecular layer, however, there are some basket cells that reside in the middle third of the molecular layer [36]. In general, basket and stellate cells are considered to be on a continuum of transitional morphology – a suggestion first put forward by [37] and therefore we consider them a single class for the purpose of the current study. Molecular layer cell firing rates were broadly similar to those of Golgi cells ranging from ∼2–35 Hz (mean ∼10 Hz) in agreement with previous descriptions by Ruigrok and co-workers [9]. Example data from a juxtacellularly labelled basket/stellate cell are shown in Figure 1E.

Our previous studies in anaesthetised rats have shown that cerebellar cortical units (granule, Golgi and Purkinje cells) commonly have distinctive response patterns following somatosensory stimulation [7], [22], thus aiding their identification as different neuronal elements [38]. For future experiments, in different cerebellar areas or in awake animals, somatosensory stimulation may not be possible thus an alternative approach for identifying cells is required. Whilst spike-shape information can be useful, particularly for Purkinje cells and mossy fibres, it is not always helpful (c.f. Golgi cell, granule cell, basket/stellate cell spike-shapes). Similarly, techniques such as juxtacellular labelling are not always possible, particularly in behaving animals, or in preparations where it is impractical to sacrifice the animal, so whilst this has proved important for validating our dataset (see Methods) we developed an approach for identifying cells using their spontaneous activity patterns. Ruigrok and colleagues [9] have made progress in this direction although their decision-tree algorithm does not provide probabilistic feedback to the experimenter. Therefore we aimed to address this shortfall by developing a method that enables the experimenter to choose their preferred confidence threshold in order to objectively accept cells for further study or reject them as unclassified units.

### Analysis of Spontaneous Firing Rate and Irregularity

We assessed a range of statistics describing firing rates, irregularities and burstiness of firing using both the arithmetic and logarithmic spike-time series. The logarithmic transformation makes the ISI distributions more symmetric [39]; for review see [40]. We computed mean, median and modal ISI, mean spike frequency (MSF) and mean instantaneous spike frequency [7], alongside a range of irregularity measures including the coefficient of variation (CV), mean CV2 (relative difference of adjacent ISIs [5], [41]), the local variation (Lv [38], [42]), the revised local variation (LvR [43]), the instantaneous irregularity (IR [44]), the geometric average of the rescaled cross-correlation of the ISIs (SI [45]), the CV of log interval series (LCV) and the log-interval-entropy (ENT [39]). The burstiness of firing was computed by the 5 th percentile of the ISIH [9]. These parameters are defined in Appendix S1.

A GPC model aimed at classifying all cell types with a combination of all 13 of our firing rate and irregularity statistics, lead to an overall accuracy of 89.4% (126/141 cells correct), although an analysis of the cell class specific errors revealed poor performance in identifying molecular layer neurones (38.1%, 8/21 correct). Considered as a whole, 24% (5/21) molecular layer neurones were misclassified as granule cells, 5 as Golgi cells and 3 as Purkinje cells. This poor performance in classifying molecular layer neurones led us to explore the performance of a molecular layer specific model. To be of use to the experimenter, layer-specific models require the experimenter to have knowledge of the cell layer being recorded from: for superficial cortical regions depth monitoring is straightforward, whilst for deeper areas electrophysiological separation of the granular and molecular layers is required, which is nonetheless eminently achievable [25].

Three of the most useful statistics for providing separation within the granular and molecular layer cell classes are plotted in Figure 2A–C & I–K; LCV, MSF and ENT, respectively. Note that ENT is expressed using a logarithmic base of 2, providing a value of bits; thus a doubling of the variance is equivalent to an increase in entropy by one bit. Considering each parameter alone, we built a GPC model (see Methods) to obtain probabilities of each unit belonging to a particular cell class. This step generates decision boundaries between cell classes (defined as equal probability of belonging to neighbouring cell classes), enabling a probabilistic classification of each neurone analysed (coloured panels, Figure 2D–F). Using the leave-one-out cross-validation (LOO-CV) technique (see Methods), both granular layer univariate models using either LCV or MSF correctly classified 72.5% of cells, whilst the ENT based model performed at 65% accuracy. As these individual parameters performed rather poorly, we therefore explored twin-variate GPC models by combining LCV-ENT, MSF-LCV and MSF-ENT. The GPC model combining LCV-ENT achieved 95% accuracy using LOO-CV (6 mistakes in 120 cells, 2 Golgi cells misclassified as Purkinje cells, 2 Purkinje cells misclassified as Golgi cells, one as a granule cell and one as a regular firing mossy fibre unit), MSF-LCV achieved 95.8% accuracy (5 mistakes in 120 cells; 3 Golgi cells misclassified as mossy fibre units, one mossy fibre unit as a Golgi and one Purkinje cell as a granule cell) although in comparison MSF-ENT achieved a much higher accuracy (99.2%) with only 1 Purkinje cell misclassified as a Golgi cell. We evaluated the statistical significance of the differences between the two selected twin-variate models (MSF-LCV and MSF-ENT). In Appendix S1 we show that the MSF-ENT model performed significantly better (significance threshold α = 0.05) than the MSF-LCV model using either a binomial distribution or a Wilcoxon ranksum test.

Following the same format for Figure 2A–H, in Figure 2I–P we show the same statistics for the molecular layer neurones and Purkinje cells. As univariate statistics, LCV, MSF and ENT offered classification accuracies of 80.5%, 92.7% and 85.4% respectively (Figure 2I–N). In the twin-variate models, MSF-LCV and MSF-ENT each achieved 92.7% accuracy (38/41) with the LOO-CV indicating that for the MSF-LCV model, 13/15 molecular layer neurones were correctly predicted with 2 misclassified as Purkinje cells, with 1/26 Purkinje cells misclassified, whereas the MSF-ENT model misclassified 2/15 molecular layer cells as Purkinje cells and 1 Purkinje cell misclassified as molecular layer cell. Although no gains in overall classification accuracy for molecular layer cells were achieved using the twin-variate models compared to MSF alone, nonetheless, the creation of two dimensional decision boundaries and probability contours aids in the process of probability-thresholding each classification decision, i.e. selecting cells with the highest probability of belonging to a particular class, and thereby leads to an overall increase in quality of the classifications made by the model.

Due to the relatively limited size of the dataset used to build this model, we reserved a further 6 molecular layer neurones (4 basket cells and 2 stellate cells) as a test dataset. When projected into the MSF-ENT model, 83% (5/6) were correctly classified with 1 stellate cell misclassified as a Purkinje cell (filled circles Figure 2P). Whilst a model that classifies only molecular layer neurones from Purkinje cells at face-value, might seem redundant, our Purkinje cell firing statistics are derived from the complete spike-trains of these cells (simple and complex spikes). Thus although many experimenters rely on observing complex spikes for definitive identification, complex spike 'visibility' is sensitive to electrode position, up- and down-states of Purkinje cells and the vigilance state of the animal [46], [47]. Therefore, the value of our approach for both granular and molecular layer models, is in removing the experimenter's reliance on complex spikes to identify Purkinje cells.

Within the cell classes, Golgi cell and Purkinje cell populations showed significant correlation between MSF and ENT (rho −0.66, p = 4.7*10^−7^; rho = −0.58, p = 0.0024, respectively; Spearman's rank correlation test) whilst neither the granule cells, nor mossy fibre units or the molecular layer cells showed a significant correlation between firing rate and irregularity. These data indicate that faster firing rates infer higher regularity of spike timing in Golgi cells and Purkinje cells.

### Entropy as a Robust Estimator of Irregularity

Although our MSF-ENT model performed to a high level of accuracy for both the molecular layer and granular layer models (92.7% and 99.2% respectively) we re-evaluated the granular layer model by substituting ENT with a variety of irregularity measures and similarly by substituting MSF with alternative measures of firing frequency. The results of this analysis using LOO-CV are shown in Figure 3A & 3B respectively, indicating that against MSF, the optimal irregularity measure to complement this is ENT, with the poorest performing combination being MSF-SI (89% accuracy) with CV slightly ahead (90.8%). The Lv and LvR perform equally well at 91.6% whilst the widely adopted measure CV2 performed as well as IR (95%) with LCV slightly behind ENT at 95.8%. In comparison, the optimal firing frequency measure to compliment ENT was MSF with modal ISI and median ISI measures each achieving 93.3% accuracy and mean instantaneous firing frequency (98.3%) offering a similar accuracy as MSF. Although we did not explore all possible parameter combinations between the frequency and irregularity measures, any gains would be marginal since MSF-ENT offers 99.2% classification accuracy. Similar substitution of the irregularity metrics in the molecular layer model offered no further improvements in classification accuracy, with all MSF-irregularity metric combinations offering 92.7% accuracy. In terms of substituting the firing frequency metrics, the ENT-mean instantaneous frequency combination decreased classification accuracy to 90.24% (37/41), with all other combinations offering no further improvement over the MSF-ENT combination (data not shown).

As the neurones in our dataset had a wide range of firing rates, and therefore variable spike numbers (minimum 62 spikes, granule cell; maximum 13560 spikes, Purkinje cell; see Methods), we investigated how spike train length affects classification accuracy. As we cannot be sure how many spikes are required to capture a neurone's full repertoire, we recomputed the granular layer MSF-ENT model using either 30 ISIs or 60 ISIs for all cells. Using 30 ISIs, prediction accuracy using LOO-CV fell to 95% (Figure 3C) with 1 granule cell being misclassified as a Golgi cell and vice-versa, and 2 Golgi cells being misclassified as regular firing mossy fibres and vice-versa. Using 60 ISIs improved performance with only 1 Golgi cell misclassified as a mossy fibre unit leading to 99.2% LOO-CV classification accuracy (Figure 3D, c.f. Figure 2H). We also followed the same approach with the molecular layer MSF-ENT model, using either 30 or 60 ISIs. In both scenarios, LOO-CV classification accuracy increased from 92.7% (38/41) to 95.4% (39/41); see Figure 3E & F. This change is attributable to a single mistakenly classified Purkinje cell (close to the decision boundary - see Figure 2P) being then correctly classified when the smaller samples of ISIs were used to compute its statistics. Thus, in the 30 ISI and 60 ISI models, 2 molecular layer neurones were incorrectly classified, representing no change from the original model using all available ISIs. In summary, our data indicate that MSF-ENT offers an optimal combination of firing pattern statistics for reliable and robust prediction of the identity of neurones using relatively small samples of activity obtained from both the granular and molecular layers.

### Comparison of GPC with Decision-tree Algorithm

Our GPC approach can be used in one of two ways - the decision boundaries can be interpreted as binary 'black and white' decisions or more powerfully, the probabilistic nature of our approach allows the experimenter to choose confidence levels for the acceptance or rejection of each individual classification. In this way, cells that fall in a parameter space, say with p<0.7 can be rejected as unknown cells. Applying our model in both modes, 'black and white' and 'shades of grey' we compare the change in classification decisions for our rat dataset in Figure 4A. For Golgi cells and granule cells, probability-thresholding pruned 4/50 Golgi cells and 1/21 granule units as unknown with the remainder of cells being 'highly likely' to belong to their particular class, thus classification accuracy for these cells remained at 100%. For the molecular layer cells, probability-thresholding pruned 3/21 units as unknown leading to an accuracy of 89% (16/18; c.f. 90% un-thresholded).

For a performance comparison, we used our datasets to calculate each of the parameters and ensuing decision-steps described in the decision-tree algorithm developed by Ruigrok and colleagues [9]. The decision-tree algorithm correctly classified 48% of Golgi cells (24/50) with 12 unknown cells (summarised in Figure 4A and Table 1), 13 Golgi cells were misclassified as unipolar brush cells and 1 as a molecular layer cell (i.e. overall 63.2%; 24/38 when disregarding unknown cells). For granule cells, 7/21 were correctly classified (33%) with 3 unknown cells and 11 misclassified as molecular layer cells (i.e. 38.9%; 7/18 when disregarding unknown cells). For molecular layer cells, the decision-tree algorithm correctly identified 61.9% (13/21) of cells, with 6 unknowns, and 1 cell misclassified as a Golgi cell and 1 cell as a granule cell (i.e. 13/15≈86.7% when disregarding border cells); our GPC model achieved a similar classification accuracy for molecular layer cells when compared to the decision-tree algorithm. In terms of probability-thresholding, our non-thresholded GPC correctly classified 100% (23/23) of regular firing mossy fibre units, with the thresholded GPC pruning 2 units as unknown, while in comparison, for Purkinje cells, the GPC misclassified 1 cell as a Golgi cell (1/26) whereas post-thresholding, 5 cells were pruned as unknown with the remaining 100% (21/21) of cells correctly identified (data not shown but see Table 1). As the decision-tree algorithm was not built to include mossy fibres or Purkinje cells and since we were not able to obtain data from identified unipolar brush cells we could neither compare nor assess, respectively, performance of our GPC with these cell types.

**Table 1:** Classification accuracy for GPC vs. decision-tree algorithm.

Cell Class	GPC (raw)	GPC (p>0.7)	Decision-tree
Rat Golgi cells (n = 50)	100	100 (n = 46)	63
Rat Granule cells (n = 21)	100	100 (n = 20)	39
Rat Basket/Stellate cells (n = 21)	90	89 (16/18)	87
Rat Mossy fibres (n = 23)	100	100 (n = 21)	n/a
Rat Purkinje cells (n = 26)	96	100 (n = 21)	n/a
***Cross-species comparison***			
Mouse Granule cells (n = 5)	80	100 (n = 4)	66
Cat Golgi cells (n = 11)	100	100 (n = 10)	100
Cat Granule cells (n = 16)	100	100 (n = 13)	64

Numbers indicated are %-accuracy calculated excluding unknown cells. Numbers in parentheses indicate sample size for each cell class; note that for the probability-thresholded GPC sample sizes decreased as cells were excluded as unknown.

Note that in our comparison of the decision-tree algorithm and our GPC models we do not include 'unknown cells', although with the selection of an arbitrary probability threshold, cells with confidence estimates below this threshold would be rejected as unknown. Therefore, considered overall (i.e. for Golgi cells, granule cells and basket/stellate cells, but excluding Purkinje cells and mossy fibres), the decision-tree algorithm correctly identifies 47.8% of cells (44/92 ) or 62.0% (44/71) when disregarding unknown cells. In comparison our models (which include Purkinje cells and mossy fibres but not unipolar brush cells) offer 92.7% and 99.2% accuracy for the molecular- and granular layers, respectively. We show that the decision-tree algorithm performs poorly in classifying Golgi cells and granule cells, despite having been designed to classify these cell types. Furthermore, we show that the probability-thresholding offered by our GPC approach leads to the pruning of cells close to the equi-probable decision boundaries between cell classes, thereby improving the overall quality of the classifications that are retained.

### Cross-species Comparison of MSF-ENT

Given the conservation of cerebellar interneurone features between species [48]–[51] we applied our GPC models to datasets obtained from laboratories examining other species. These included identified granule cells recorded in the paraflocculus of anaesthetised mice [24], identified granule cells and Golgi cells recorded in lobule IV/V in decerebrate cats [10]. Projecting the mouse granule cells into the granular layer MSF-ENT model yielded 80% accuracy (4/5 cells correct) with the mistaken cell lying close to the granule cell - Golgi cell decision boundary (Figure 4B, cell entropy = 7.34, decision boundary entropy = 7.41, probability [Golgi cell class] = 0.57). Similarly, for the identified cat cells our model yielded 100% accuracy (Figure 4C; inset panels; 27/27 cells correct). These findings indicate that the rat-based decision boundaries generalise to mice and cats, at least for granule cells and Golgi cells.

Assessing our awake animal datasets represents an ideal benchmark since neither the decision-tree algorithm nor our own models were built using these datasets. In this regard, the decision-tree algorithm generally performed with lower accuracy in the anaesthetised mouse and decerebrate cat preparations (Table 1 & Figure 4D; c.f. rat data Figure 4A). Our approach thus holds the promise of offering experimenters a means of standardising the classification of neurones between laboratories and across species, with a probabilistically determined means of accepting/rejecting each individual classification.

### Application to Awake Animal Preparations

Although our model performs well in classifying neurones in anaesthetised and decerebrate preparations, ideally its most powerful application would be in the classification of neurones in awake animals, where the opportunities for obtaining ground-truth identification are often severely limited. To this end, we assessed a variety of datasets composed of granular layer neurones, which we would expect to include large interneurones such as Golgi cells, along with Purkinje cells recorded in lobule HVI in awake rabbits [25], the parafloculus in awake rhesus monkeys (unpublished data) and the nodulus uvula in awake rhesus monkeys (unpublished data). In these datasets, where ground-truth was not available (with the exception of Purkinje cells), we applied our model to probabilistically identify cells that fall within a particular MSF-ENT parameter space, i.e. the most Golgi cell-like units.

**Figure 5:**
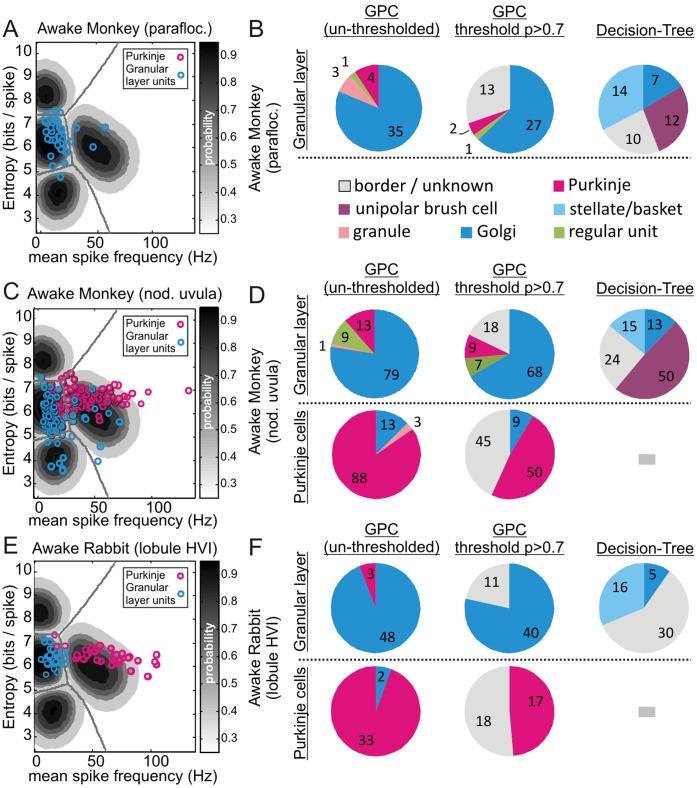
Putative classification of neurones in awake monkeys and rabbits. **A, C** & **E** re-plot our GPC decision boundaries and probability contours, in each case with a selection of granular layer units (blue circles), from the ventral parafloculus of awake monkeys (**A**), alongside Purkinje cells in the nodulus uvula of awake monkeys (**B**) and alongside Purkinje cells in the lobule HVI of awake rabbits (**E**). Besides each plot, the pie charts (**B**, **D**, **F**) illustrate the classification decisions arising from the un-thresholded GPC (*left* column), the p>0.7 thresholded GPC (*middle*) and for comparison, the decision-tree algorithm (*right*) for both the granular layer units and where appropriate, the Purkinje cells (i.e. decision-tree algorithm cannot deal with Purkinje cells). For the granular layer, all classifications remain putative and in this setting the GPC highlights cells with, for example, the most Golgi-like firing patterns. In comparison, the decision-tree algorithm in all cases suggests that a subset of the granular layer neurones are molecular layer cells (c.f. Figure 5B, 5D & 5F).

Data from ventral parafloculus in awake rhesus monkeys consisted of 43 granular layer cells (Figure 5A). The GPC model suggested that 35 of these units (81%) bore a close correspondence to identified Golgi cells (Figure 5B). Applying an arbitrary probability threshold (p>0.7) pruned 13 cells as unknown, leaving 27/30 (90%) units as being highly likely to be Golgi cells (unknown cells excluded). In comparison, the decision-tree algorithm suggested 7 units as Golgi cells (21% - when unknowns are excluded).

Alternative data from the nodulus uvula in awake rhesus monkeys, consisting of 102 granular layer units and 104 Purkinje cells were analysed in the same way (Figure 5C), with the GPC suggesting 79/102 (77%) candidate Golgi cells, pruned to 68/84 (81% - unknowns excluded) following probability thresholding. In comparison, the decision-tree algorithm suggested 13/78 (17%) candidate Golgi cells. In terms of Purkinje cells, our model correctly classified 88/104 (85%; un-thresholded) and 50/59 (85%) following probability-thresholding with 45 Purkinje cells pruned as unknown (Figure 5D). The nodulus uvula dataset also included 3 non-Purkinje cell units recorded in the molecular layer. Our molecular layer model misclassified these as Purkinje cells whilst the decision-tree algorithm suggested 2 of these units were stellate/basket cells and the remaining unit was a unipolar brush cell.

Finally, we assessed data obtained from awake rabbits containing 35 Purkinje cells and 51 granular layer units (Figure 5E). Our model suggested 48/51 candidate Golgi cells, with thresholding pruning 11 units as unknown, leaving 40/40 units (100%) considered highly likely to be Golgi cells. The decision-tree algorithm suggested 5/21 (24% - unknowns excluded) candidate Golgi cells. In terms of Purkinje cells, the GPC correctly classified 33/35 Purkinje cells (94%) with probability-thresholding identifying 17 units as highly likely to be Purkinje cells (100%) with 18 unknowns (Figure 5F).

In summary, we show how our GPC approach can highlight subsets of cells bearing the closest similitude to identified cell types, thereby enabling the experimenter to probabilistically accept or reject units for further study based on the likelihood of their belonging to a particular cell class. Although our GPC model is derived from firing patterns obtained in anaesthetised rats, the choice of parameters and associated decision boundaries nonetheless appear to generalise well across species and from anaesthetised to awake animal preparations.

## Discussion

Obtaining accurate identification of neurones recorded in awake animals represents a major barrier to systems and network-level neuroscience experiments. We have approached this problem in the cerebellar cortex by developing a probabilistic model built with data from identified cells. We show that our built GPC model accurately predicts cellular identity across species in mice, rats and cats and offers statistical inference of cell type for putatively classified cells in awake primates and rabbits. We further show that our probabilistic models provide robust classification using easily obtainable measures of spontaneous activity derived from short excerpts of data (60 inter-spike intervals) enabling classification decisions to be obtained within rapid experimental-time frames (a few seconds on average) offering the chance to influence experimental decision-making in awake animal preparations. Finally, our approach may facilitate inter-laboratory comparison of datasets by offering a statistical means of accepting/rejecting cells for further study.

Differentiation between cerebellar cortical cell types using established criteria usually relies on qualitative descriptions of cell firing patterns which are not easy to generalise. Whilst the decision-tree algorithm used by Ruigrok and colleagues (2011) makes progress toward solving this problem, comparison with our own cross-species datasets indicates their criteria do not generalise to preparations beyond their own as the decision-tree algorithm offered relatively poor classification accuracy for our identified Golgi cells and granule cells in mice, rats and cats (see Figure 4 and Table 1). This may reflect unique characteristics of the cells they analysed within the particular cerebellar cortical compartments in the rat (primarily ventral uvula and nodulus) and flocculus) and rabbit (flocculus). Alternatively, other factors may account for this mismatch; in the present study, we built our models with a considerably larger dataset than Ruigrok *et al*. and our test data was not used to build our GPC models whereas their approach is weakened by using the same data to test their model as was used to build it. It is well known from the machine learning literature that this is a poor indicator for the generalization performance on unseen data due to the problem of over-fitting [52]. Furthermore, the decision-tree classification accuracies represent only how well cell types can be distinguished by drawing orthogonal decision lines that ignore any parameter correlations, and as we show for some cell types (Golgi cells and Purkinje cells), firing rate and irregularity can be correlated. These factors may in part explain why the decision-tree algorithm achieved low performances on our datasets.

Unfortunately, due to scarcity/absence of reported recordings from some of the rarer/harder to obtain types of cerebellar neurones we were not able to assess data from unipolar brush cells, Lugaro cells, globular cells and other types of mossy fibre units, which can have firing patterns different from our regular-firing mossy fibres [6], [12], [23], [32]. Further, not all cells are spontaneously active thereby precluding their inclusion in our analysis; many granule cells are inactive at rest *in vivo,* nonetheless this can aid in their identification [6], [9]–[11], [24]. We still rely in part on spike-shape information for the identification of mossy fibre terminals [4], [10], [32], [35]. However, our method does not require convoluted quantitative measurement of spike-shapes [14]–[16] thereby reducing the analysis burden and avoiding non-trivial comparisons between laboratories/measurement style. In this regard, our GPC model can accurately distinguish Purkinje cells from other cell types without the need for the experimenter to detect/assess complex spike discharges which are known to be labile [46], [47]. Although our approach includes Purkinje cells and mossy fibres, which were not examined by Ruigrok *et al.* [9], Purkinje cells can be incorporated in to the decision-tree algorithm as shown by Hensbroek *et al.* [53] although the parameters for this new arm of the algorithm were not available to us; using our own data we found that in its published form the decision-tree algorithm near uniformly misclassifies Purkinje cells as stellate/basket cells.

The success of our approach relies on combining two easily computed parameters - mean spike frequency and spike-train irregularity measured using log-interval-entropy. Log-interval-entropy has been previously employed to measure neuronal activity in the neuroendocrine system [54]–[57] and in the subthalamic nucleus of Parkinsonian patients [58]. Logarithmic transformation of inter-spike intervals affords several advantages; firstly it reduces the effect of long-ISIs on the standard deviation, thus unlike the CV, entropy does not weight long-ISIs at the expense of short-ISIs due to the inherent asymmetry in ISI distributions (since CV is sensitive to ISIH skewness). Secondly, entropy also provides accurate estimates of irregularity with small data sets [54], [59], in our case producing reliable models using 60 ISIs. Thirdly, entropy is independent of the units used to measure time, for example arbitrary time-binning used to measure firing rate, thus for individual cells it quantifies firing irregularity in a manner statistically independent from firing rate. For a Poisson process, the log-interval-entropy is also independent of frequency because it is constant (∼7.9 bits using a 0.02 ln time resolution [39]), thus entropy also measures the maximal amount of information that each spike may encode [60]. In this regard, it is noteworthy that average entropy values for granule cells were 8.09±0.08 bits/spike (rats: mean ± standard error; see Figure 2H) thus these cells, via their interaction with excitatory mossy fibres and inhibitory Golgi cells, may be optimised for maximal coding capacity [23]. In contrast, average Purkinje cell entropy (rats: 5.92±0.1 bits/spike), Golgi cell entropy (rats: 6.30±0.08 bits/spike) and molecular layer cells (rats and cats: 7.1371±0.61 bits/spike; see Figure 2P) suggest overall lower coding capacities per spike. This might arise through the considerable synaptic convergence these cell types receive from granule cells [61]. Furthermore, Purkinje cells and Golgi cells have auto-rhythmic spike-generators leading them to be spontaneously active *in vitro* [62], [63] and it is likely that this serves to 'regularise' spike-timing, producing lower entropy values; a perfect metronome has zero entropy. This may also partly account the strong correlation between firing rate and entropy observed for Purkinje and Golgi cell populations which suggests the more potent influence of the refractory period may limit spike-timing at higher rates (c.f. negative rate-CV relationship [64], [65]); in this regard Golgi cells have prolonged spike after-hyperpolarisations [63].

Our cross-species comparisons highlight the broad relevance of our criteria to a variety of animals. In part, this may arise due to a degree of conserved homology between species [48]–[51] and in part due to our use of urethane anaesthesia which is reported to act via background potassium leak channels thus leaving synaptic transmission intact [66], [67] and therefore, to some extent, perhaps mimics the non-anaesthetised state; in this regard, the decerebrate cat data showed a striking correlation with the rat data. None-the-less, our mouse granule cells were recorded under ketamine-xylazine [24] which depresses granule cell transmission [68] but despite this our model nonetheless captures the key features of granule cell spontaneous activity. Considering the more challenging datasets obtained from awake primates and awake rabbits, no ground-truth was obtained for the non-Purkinje cells and our approach requires that experimenters delineate cell layers within the cerebellar cortex, however this is relatively straight-forward since the molecular layer is characterised by the presence of climbing fibre signals which can be observed in the local-field-potential signal in both anaesthetised and awake animals [25], along with monitoring the crossing of Purkinje cell layers [5], [7], [22], [28], [69] recordings can be attributed to the granular layer with a high degree of confidence. In both awake primate and rabbit preparations there is a general increase in mean spike frequency for all cell types (right-ward shift in datasets), consistent with some degree of activity suppression under anaesthesia/decerebration (c.f. Figure 2H & Figure 5A, 5C & 5E), thus re-building of the GPC model incorporating the identified Purkinje cells would likely improve the positioning of the decision-boundaries and thus robust statistical grouping of cells. Nonetheless, probability-thresholding aids in reducing the 'blur' between granular layer cells and Purkinje cells in our awake monkey data (see Figure 5C & 5D) and so although the absolute decision boundaries may not be optimal, their tolerance is increased by thresholding thus our model was able to highlight those cells bearing the highest similarity to identified cell classes and in this way enable the experimenter to choose their preferred level of confidence in their classifications. We envisage that this approach will enable experimenters to standardise acceptance/rejection of cells for further study and facilitate comparisons between datasets generated in different laboratories.

Our GPC approach can be applied and tested in a variety of other brain areas, particularly where ground-truth datasets are available: the emergence of datasets of optogenetically identified neurones in vivo will substantially facilitate efforts in this direction, see e.g. [70], as will the advent of methods for accomplishing the gold-standard ground-truth of juxtacellular labelling in freely behaving animals [71]. The advantages of our GPC approach make it highly attractive as a future research tool particularly as mean spike frequency and entropy can be calculated on a spike-by-spike basis in near real-time providing an online probabilistic cell classification which in turn could influence experimental decision-making and guide future brain-machine interfaces where real-time single neurone classification is required when decoding system- or network-level activity.

## Supporting Information

Appendix S1 **Equations for firing rates, irregularity and burstiness and hypothesis testing of GPC model parameter choice.**
(DOC)
