# Supplementary material for: Probabilistic Identification of Cerebellar Cortical Neurones across Species
Source: PLoS One. 2013 Mar 4;8(3):e57669. doi: 10.1371/journal.pone.0057669 (PMC3587648; doi:10.1371/journal.pone.0057669)
Supplement: Appendix S1 — Equations for firing rates, irregularity and burstiness and hypothesis testing of GPC model parameter choice. (DOC) [file pone.0057669.s001.doc]

**Appendix S1**

**Firing rates, irregularities and burstiness of firing**

Suppose we have:

$$\begin{matrix} \text{n + 1 spikes,} \\ \text{n interspike intervals (I),} \\ t_{i}=time\text{ of occurence for spike i,} \\ \text{I}_{\text{i}}=\text{ length of 'i' th inter-spike interval.} \end{matrix}$$

**Mean Spike Frequency**

The mean spike frequency or the average firing frequency is the number of inter-spike intervals (ISI’s) divided by time difference between the first and the last spike:

$MSF=\frac{number\text{ }of\text{ }spikes-1}{t_{n+1}-t_{1}}=\frac{n}{t_{n+1}-t_{1}}=\frac{n}{\sum_{i=1}^{n} \text{I}_{\text{i}}}$.

The time difference between the first and the last spike, t_n+1_ – t_1_, can be calculated as the sum of the n inter-spike intervals $\sum_{i=1}^{n} \text{I}_{\text{i}}$.

**Mean Instantaneous Frequency**

The mean instantaneous frequency is the average of instantaneous frequencies $\frac{1}{I_{i}}$:

$mean\text{ inst. freq.}=\frac{1}{n}\sum_{i=1}^{n} \frac{1}{I_{i}}$.

**Median ISI**

Suppose the inter-spike interals I_i_ are sorted in ascending order, i.e. I_1_ ≤ I_2_ …≤ I_n_ . The median ISI of a series of n ISI’s is defined as:

$$\begin{matrix} IS\mathrm{I}_{\text{median}}=ISI_{\frac{n}{2}+0.5}\text{ for odd n,} \\ \text{ISI}_{\text{median}}=\frac{ISI_{\frac{n}{2}}+ISI_{\frac{n}{2}+1}}{2}\text{ for even n} \end{matrix}$$

**Mode of ISI**

Suppose the probability distribution of the inters-pike intervals is given by:

p(I), with I the variable denoting the inter-spike intervals. The mode of the ISI (inter-spike intervals) is then given by the most likely ISI:

$IS\mathrm{I}_{\text{mode}}=\mathrm{argmax}p(I)$.

**5 th percentile of ISI**

Suppose the probability distribution of the inters-pike intervals is given by:

p(I), the 5 th percentile is the ISI interval for which 5% of the inter-spike intervals is smaller:

$p(I<I_{5\%})=0.05$.

In practice the 5^th^ percentile is estimated from n ISI’s as follows. Suppose the inter-spike interals I_i_ are sorted in ascending order, i.e. I_1_ ≤ I_2_ …≤ I_n_.

1. The sorted ISI intervals are taken as the 100*(0.5/n), 100*(1.5/n),..., 100*((n-0.5)/n) percentiles.
2. Linear interpolation is used to compute percentiles for percent values between 100*(0.5/n) and 100*((n-0.5)/n).
3. The minimum or maximum ISI’s are assigned to percentiles for percent values outside that range.

The 5 th percentile can capture the burstiness of the firing of a neuron, a low 5 th percentile for a given firing rate indicates more burstiness.

**Entropy of the ISI distribution of a spike train**

First the natural logarithm of the ISI’s given in ms is taken. Subsequently a histogram of the log_e_ ISI is computed using a binwidth of 0.02 log_e_(time). Next, a Gaussian convolution is employed [40] to mitigate the effects of an arbitrary choice of bin width. We used a kernel, with a standard deviation one-sixth of the standard deviation of the log ISI’s, and a range from −3 to +3 standard deviations [39].

The entropy of the log of the ISI histogram p(I_i_) with N bins is then computed as:

$Ent(I)=-\sum_{i=1}^{N} p(I_{i})\log_{2}p(I_{i})$.

The entropy captures the randomness of the ISI’s from a spike train. A low entropy means that a neuron fires regularly, while a large entropy indicates a neuron fires more irregularly.

**CV2: average coefficient of variation for a sequence of 2 ISI’s**

The C_v2_ value between 2 consecutive ISI’s is the ratio of their standard deviation to their average, multiplied by a factor $\sqrt{2}$.

$C_{v2}=\sqrt{2}\frac{\left|I_{i+1}-I_{i}\right|/\sqrt{2}}{\left(I_{i+1}+I_{i}\right)/2}=\frac{2\left|I_{i+1}-I_{i}\right|}{I_{i+1}+I_{i}}$.

The average C_v2_ is obtained as:

$\left\langle C_{v2}\right\rangle=\frac{1}{n-1}\sum_{i=1}^{n-1} \frac{2\left|I_{i+1}-I_{i}\right|}{I_{i+1}+I_{i}}$.

Multiplaction by a factor $\sqrt{2}$ results in an average C_v2_ of 1 for a Poisson process, see [41].

An average of C_v2_ over all consecutive pairs of ISI’s estimates the intrinsic variability of a spike train, nearly independent of slow variations in average rate, by comparing adjacent intervals and not widely separated intervals [41].

**CV: coefficient of variation**

C_v_ is the standard deviation of the ISI’s divided by the average ISI.

$C_{v}=\frac{std\left(I\right)}{\left\langle I\right\rangle}$.

However, it has to be noted that the coefficient of variation, will not give an accurate estimate of the variability of a neuron when the mean rate changes over time. The C_v_ compares ISI’s that come from different mean rates [41].

**IR: Instantaneous irregularity**

The instantaneous irregularity computes the average of the absolute difference between the natural logarithms of 2 consecutive ISI’s.

$IR=\frac{1}{n-1}\sum_{i=1}^{n-1} \left|\log_{e}\left(\frac{I_{i+1}}{I_{i}}\right)\right|$.

**Lv: Local variation**

The summand in the local variation is proportional to the squares of the individual terms of C_V2_:

$L_{v}=\frac{3}{n-1}\sum_{i=1}^{n-1} \left(\frac{I_{i}-I_{i+1}}{I_{i}+I_{i+1}}\right)^{2}=\frac{3}{n-1}\sum_{i=1}^{n-1} \left(1-\frac{4I_{i}I_{i+1}}{\left(I_{i}+I_{i+1}\right)^{2}}\right)$.

The factor 3 was chosen so that the average Lv value is equal to 1 for a Poisson process [38].

**LvR: Revised local variation**

Because Lv, SI, C_v2_, and IR are still somewhat dependent on firing rate fluctuations. The Lv is adapted to a revised Lv, LvR. Assuming that rate dependence is caused by the refractory period, R, that follows a spike, the refractoriness is compensated for in the LvR [43]:

$L_{vR}=\frac{3}{n-1}\sum_{i=1}^{n-1} \left(1-\frac{4I_{i}I_{i+1}}{\left(I_{i}+I_{i+1}\right)^{2}}\right)\left(1+\frac{4R}{I_{i}+I_{i+1}}\right)$.

In the experiments we set the refractory period to 1 ms.

**LcV: coefficient of variation of the log ISI**

The coefficient of variation of the log ISI is similar to the CV, but with the log taken of the ISI’s.

$$LcV=\frac{std\left(\log(I)\right)}{\left\langle\log(I)\right\rangle}$$

**SI: Geometric average of the rescaled cross-correlation of ISI’s**

This S_I_ index computes the average of the logarithm of the ratio of the geometric mean and the arithmetic mean. The arithmetic mean is always larger or equal to the geometric mean and they are only equal if I_i_ and I_i+1_ are equal. The lower bound for S_I_ is hence equal to 0 when all ISI’s are equal.

$$S_{I}=-\frac{1}{n-1}\sum_{i=1}^{n-1} \frac{1}{2}\log\left(\frac{4I_{i}I_{i+1}}{\left(I_{i}+I_{i+1}\right)^{2}}\right)$$

**Hypothesis testing to test whether the MSF-ENT model performs better than the MSF-LCV model**

We follow 2 approaches to test whether the MSF-ENT model using leave-one out cross validation (LOO-CV) gives statistically significant higher values than the MSF-LCV model.

In the first approach we assume that predicting the cell type of the 120 cells using a leave-one out cross validation is a series of 120 (N) Bernouilli trials. A success is defined as a correct prediction of the cell type by the model and a failure as an incorrect prediction of the class label. A series of N independent Bernouilli trials in which the random variable is the sum of successes follows a binomial distribution B(N,p), with N the number of trials and p the probability of success in each trial. Strictly speaking, the number of correct decisions of the leave-one-out cross validation will not follow a binomial distribution. Firstly, the trials are not really independent as cells with similar parameters are likely to get the same class label. Secondly, when the same cell is presented multiple times to the LOO-CV, the model will almost always invariantly predict the same cell type for the cell, while there should in fact be a probability 1-p to make an error. Nevertheless, we will use the binomial distribution as a first approximation.

We test whether the 119 correct decisions of the MSF-ENT model are statistically significantly larger than what can be expected from the MSF-LCV model. The parameters of the binomial distribution for the LOO-CV are N = 120 trials and the success rate is 115/120. These parameters fully characterize the binomial distribution with probability mass function f(x,N,p). The probability to achieve a value higher or equal than 119, i.e. 119 or 120, is f(119,120,115/120) + f(120,120,115/120) = 0.03158 + 0.00605 = 0.03763. Hence, we can conclude that the MSF-ENT model performs statistically significantly better than the MSF-LCV model using a significance level α equal to 0.05.

In the second approach we simulate the procedure of a LOO-CV N times, both for the MSF-ENT and the MSF-LCV model, and apply a non-parametric statistical test to assess whether the performance of the LOO-CV under the MSF-ENT model is significantly higher than that of the MSF-LCV model. This procedure works as follows. For both the MSF-ENT and the MSF-LCV selection of parameters we perform N-times a LOO-CV on N-1 cells. Within one LOO-CV N-2 cells are used to train a model and 1 cells is used to test the accuracy and this is iterated over all N-1 cells. All cells are left out once over all LOO-CV’s, hence leading to 120 LOO-CV’s. This leads to 120 performances of the LOO-CV for both the MSF-ENT and the MSF-LCV model. We apply the Wilcoxon rank sum test in order to test whether the MSF-ENT model gives systematically larger performances than the MSF-LCV model. The median performance over 120 LOO-CV’s of size N-1 for the MSF-ENT and the MSF-LCV model are 99.16 % and 95.80 % respectively. A right-tailed Wilcoxon ranksum test leads to a p-value < 10^-3^, showing that the MSF-ENT leads systematically to significantly better performances than the MSF-LCV model under the α = 0.05 significance level.
